# Malpositioning of a Central Venous Catheter in the Vertebral Vein Through the Cervical Transverse Foramen: A Case Report

**DOI:** 10.7759/cureus.76637

**Published:** 2024-12-30

**Authors:** Sayaka Ito, Yoshinori Maki, Naoki Hatsuda

**Affiliations:** 1 Department of Neurosurgery, Kohka Public Hospital, Kohka, JPN; 2 Department of Neurosurgery, Hamamatsu University School of Medicine, Hamamatsu, JPN; 3 Department of Neurosurgery, Ayabe Renaiss Hospital, Ayabe, JPN; 4 Department of Neurosurgery, Hikone Chuo Hospital, Hikone, JPN

**Keywords:** arteriovenous fistula, central vein, hemorrhage, malposition, misplacement, thromboembolism, vertebral artery

## Abstract

Central venous catheters (CVCs) are commonly used for multiple clinical purposes. The internal jugular vein (IJV) is preferred among the most frequently used insertion sites due to its higher success rates and lower complication risks. Although CVC placement is generally considered a safe procedure, several complications have been reported. Catheter malpositioning, one of the most common procedural complications, can lead to potentially fatal outcomes if not promptly addressed. Although catheter malpositioning is well-documented in various anatomical locations, accidental CVC cannulation of the vertebral vein is rare and has not been previously reported. We present a case of a 65-year-old man diagnosed with Guillain-Barré syndrome who underwent echo-guided CVC insertion into his right neck. The subsequent X-ray and CT scans revealed that the CVC insertion point was dorsolateral to the right sternocleidomastoid muscle. The catheter was passed dorsally to the right IJV and common carotid artery, passing through the C6 transverse foramen. The catheter's position raised concerns for potential complications, such as a vertebral arteriovenous fistula (AVF) or venous thromboembolism. Consequently, the CVC was removed after confirming the absence of AVF, bleeding, or thromboembolisms. This case is clinically significant, highlighting the potential for CVC malposition in the vertebral vein. It underscores the importance of careful monitoring during CVC insertion and removal, with particular attention to the potential for unexpected hemorrhagic events.

## Introduction

Central venous catheters (CVCs) are commonly used for multiple clinical purposes, including drug administration, central venous pressure monitoring, renal replacement therapy, total parenteral nutrition, and transvenous cardiac pacing [1]. Among the preferred sites for CVC placement, the internal jugular vein (IJV) is favored due to its higher success rates and relatively lower complication rates [2]. In clinical practice, the right IJV is often preferred because of its larger diameter, more direct route to the superior vena cava (SVC), absence of the thoracic duct, and reduced complications [3]. Although CVC placement is generally considered safe, several complications are still reported [4,5]. Catheter malpositioning, a common procedural complication, has been documented in various anatomical locations and can potentially lead to fatal outcomes if not promptly addressed [5-9]. However, to the best of our knowledge, accidental CVC cannulation of the vertebral vein has not been previously reported.

Herein, we present a rare case of CVC malpositioning in the vertebral vein through the right C6 transverse foramen, followed by successful removal, and discuss a strategy for safe CVC removal.

## Case presentation

**Figure 1:**
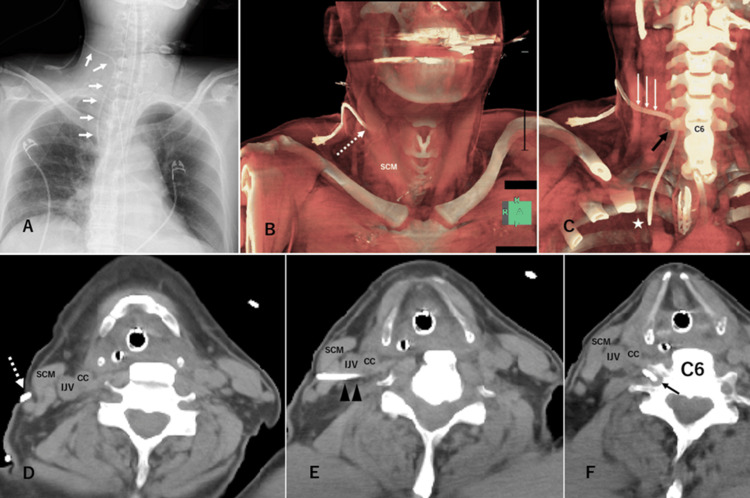
Radiological findings after the insertion of a central venous catheter (A) X-ray image and (B, C: three-dimensional-reconstructed images; D-F: axial images) computed tomography images after the insertion of a central venous catheter. The central venous catheter (CVC; white arrows in A and C) was inserted from the posterior limb of the right sternocleidomastoid muscle (SCM) (dotted arrow in D), dorsally passing (black arrowheads) the right internal jugular vein (IJV) and common carotid artery (CC) and entering the transverse foramen of C6 (black arrow in C and F). The CVC tip (white star in C) is positioned in the right brachiocephalic vein.

A 65-year-old man diagnosed with Guillain-Barré syndrome was admitted to our hospital and started on intravenous immunoglobulin infusion therapy. Despite treatment, the patient developed respiratory failure, requiring intubation and assisted ventilation. A triple-lumen CVC was concurrently inserted into the right side of the patient’s neck. The procedure was uneventful, with no technical difficulties reported. Reverse venous flow from the catheter was performed as usual. However, the postprocedural radiograph revealed an unusual catheter pathway, which did not align with the expected route of the IJV (Figure 1A).

**Figure 2:**
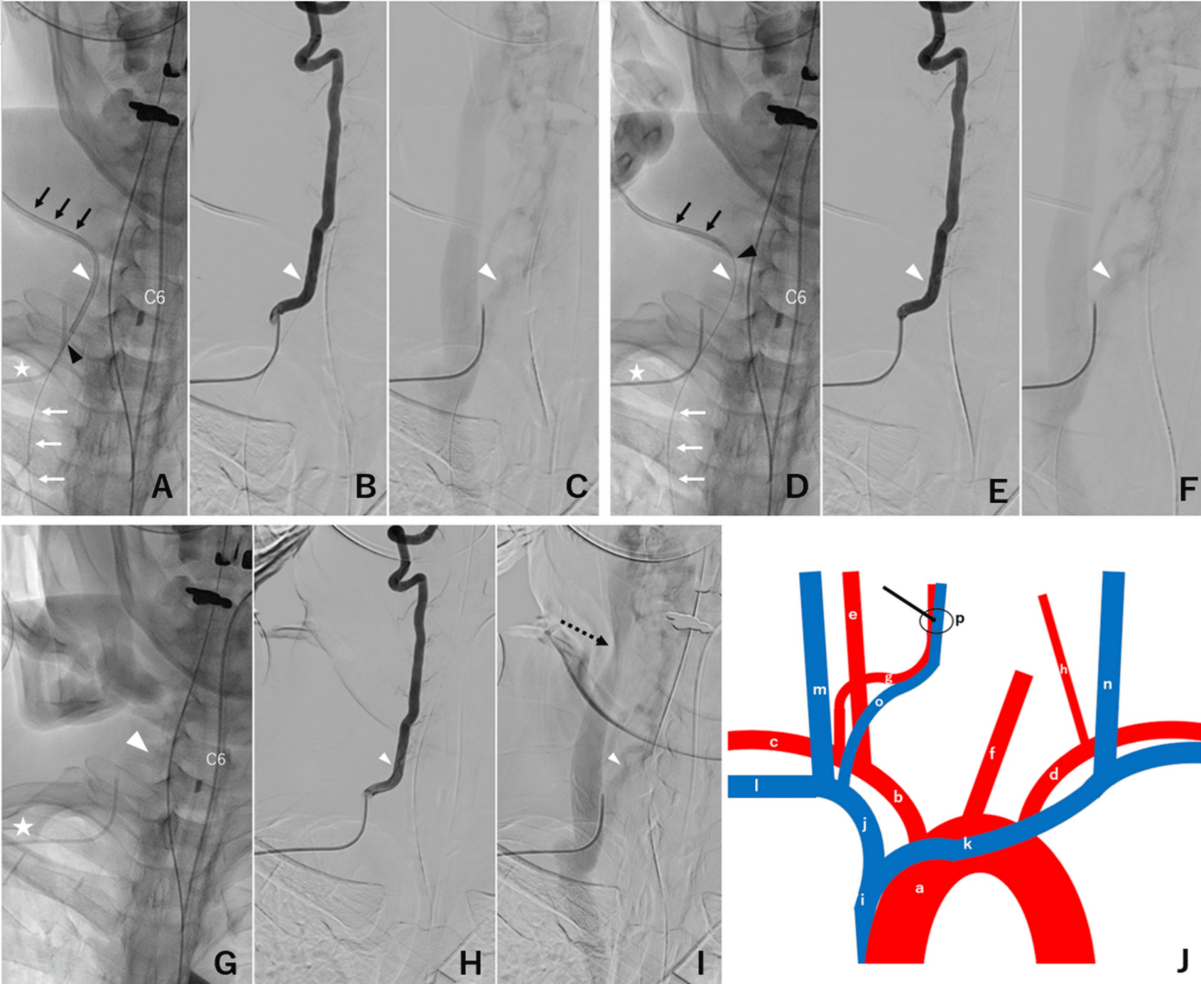
Angiographic findings and a schema during the removal of the malpositioned central venous catheter (A, D, G) X-ray images, (B, E, H: arterial phase; C, F, I: venous phase) digital subtraction angiography, and (J) angioarchitecture schema.  (A-C) Pre-, (D and F) intra-, and (G-I) post-removal of the central venous catheter (CVC) showing the guidewire (white arrows in A and D), the CVC (black arrows in A and D), the CVC tip (black arrowhead in A and D), right C6 transverse foramen (white arrowhead in A-I), a right vertebral arterial catheter (white star in A, D, and G), and a compressed right internal jugular vein (black dotted arrow). Pre-removal angiography showed no arteriovenous (AV) shunt or contrast extravasation and revealed the entry site at the vertebral vein running adjacent to the C6 transverse foramen and the C6 transverse foramen containing the vertebral artery and vein. Intra-removal angiography showed no AV shunt or contrast extravasation immediately after the removal of the CVC from the C6 transverse foramen. Post-removal angiography showing no AV shunt or contrast extravasation. Schematic drawing showing angioarchitecture (a, aortic arch; b, right brachiocephalic artery; c, right subclavian artery; d, left subclavian artery; e, right common carotid artery; f, left common carotid artery; g, right vertebral artery; h, left vertebral artery; i, superior vena cava; j, right brachiocephalic vein; k, left brachiocephalic vein; l, right subclavian vein; m, right internal jugular vein; n, left internal jugular vein; o, right vertebral vein; p, right C6 transverse foramen. Black arrow, venous puncture site in J).

Neck and chest CT without contrast enhancement confirmed that the CVC insertion point was dorsolateral to the right sternocleidomastoid muscle (Figure 1B). The catheter was passed dorsally to the right IJV and common carotid artery, passing through the C6 transverse foramen (Figures 1C-1F). A neurologist consulted the patient, with a neurosurgeon requesting the management of a malpositioned CVC. Removal of the catheter was deemed necessary, as its location in the C6 transverse foramen posed a risk of vertebral arteriovenous fistula (AVF) and venous thromboembolism. To facilitate removal, digital subtraction angiography guidance was planned in case emergent embolization was required. A 0.012-inch guidewire was inserted into the main lumen of the CVC. We cannulated a 4-F catheter through the right radial artery into the patient’s right vertebral artery (VA) (Figure 2A).

The arterial phase of the right vertebral arteriography (VAG) did not show apparent AVFs, contrast extravasations, or arterial stenoses (Figure 2B). In the venous phase of the VAG, the entry point of the CVC into the right vertebral vein, adjacent to the C6 transverse foramen, was identified (Figure 2C). Based on these radiological findings, we decided to perform transvenous embolization after catheter removal, if necessary. Subsequently, the catheter was gently retracted, leaving the guidewire intact until the catheter tip passed through the entry site (Figure 2D). A repeat right VAG showed no AV shunt, contrast extravasation, or steno-occluded vessels (Figure 2E and Figure 2F). The entire catheter and guidewire were removed, and manual compression was applied to achieve hemostasis. A right VAG performed 15 minutes after CVC removal revealed no evidence of contrast extravasation (Figures 2G-2I). The patient tolerated the entire procedure well.

## Discussion

We report a case of a CVC malpositioned in the vertebral vein through the transverse foramen. The catheter was successfully removed after careful endovascular preparation to prevent intraoperative bleeding and the risk of iatrogenic vertebral AVF formation.

Catheter malposition is one of the most common complications of CVC insertion, occurring in approximately 3%-8% of cases [10]. Several studies have revealed the advantages of ultrasound-guided cannulation over traditional anatomical landmark-based procedures [11]. However, complications can still arise [6-9]. Notably, CVC malpositioning may cause fatal complications such as cardiac tamponade, pleural infusion, and mediastinal hematoma [5]. Therefore, once identified, the malpositioned catheter should be removed as safely as possible to avoid further complications [5]. The removal strategy depends on the catheter's location.

To the best of our knowledge, only one prior case of intravenous CVC malposition has been reported in the literature [12]. In that report, a CVC was inserted into the patient’s right IJV and terminated at the right marginal sinus. Neurosurgeons anticipated and prepared for possible extravasation and emergency endovascular embolization after the catheter was removed. However, the CVC was removed without incident, and the authors recommended that intracranially positioned CVCs be removed under angiographic guidance [12].

In our case, ultrasound-guided CVC insertion was performed as recommended. However, the operator may have overlooked the abnormal skin insertion of the catheter and vertebral vein puncture. Radiography, CT, and visual examination revealed abnormal insertion of the catheter.

## Conclusions

We believe that our case study is clinically important as it highlights the potential impact of the bony structure of the transverse foramen, the angioarchitecture of the vertebral vein, and the VA on CVC insertion. After catheter placement, movement within the transverse foramen may have been restricted due to the bony structure, vertebral vein, VA, and the CVC itself. This restriction could increase the risk of malpuncture, potentially leading to a vertebral AVFs and thrombosis, which may result in thromboembolism. Although no hemorrhage or thrombosis occurred in our patient, precautions were necessary to prevent emergent embolization and thromboembolisms.

In cases of unusual medial positioning of the CVC on postprocedural radiography, catheter malpositioning should be suspected. Further studies and diagnostic tests are required to prevent secondary complications. If the catheter is placed in the transverse foramen, precautions to prevent vertebral AVF should be taken during removal.
